# Reply to Ruhl and Craig: Assessing and governing extreme climate risks needs to be legitimate and democratic

**DOI:** 10.1073/pnas.2217404119

**Published:** 2022-11-29

**Authors:** Luke Kemp, Chi Xu, Joanna Depledge, Kristie L. Ebi, Goodwin Gibbins, Timothy A. Kohler, Johan Rockström, Marten Scheffer, Hans Joachim Schellnhuber, Will Steffen, Timothy M. Lenton

**Affiliations:** ^a^Centre for the Study of Existential Risk, University of Cambridge, Cambridge CB2 1SB, UK; ^b^Darwin College, University of Cambridge, Cambridge CB3 9EU, UK; ^c^School of Life Sciences, Nanjing University, Nanjing 210023, China; ^d^Cambridge Centre for Environment, Energy and Natural Resource Governance, University of Cambridge, Cambridge CB2 3QZ, UK; ^e^Center for Health and the Global Environment, University of Washington, Hans Rosling Center for Population Health, Seattle, WA 98195; ^f^Future of Humanity Institute, University of Oxford, Oxford OX2 0DJ, UK; ^g^Department of Anthropology, Washington State University, Pullman, WA 99164-4910; ^h^Santa Fe Institute, Santa Fe, NM 87501; ^i^Cluster of Excellence ROOTS, Christian-Albrechts-Universität, Kiel 24118, Germany; ^j^Potsdam Institute for Climate Impact Research, Telegraphenberg, Potsdam 14473, Germany; ^k^Department of Environmental Sciences, University of Wageningen 6708PB, the Netherlands; ^l^Earth System Science Department, Tsinghua University, Beijing, China; ^m^Fenner School of Environment and Society, The Australian National University 2601, Australia; ^n^Global Systems Institute, University of Exeter, Exeter EX4 4QE, UK

We thank Ruhl and Craig for their letter “Designing extreme climate change scenarios for anticipatory governance” (1) in response to our manuscript “Climate Endgame” (2). We agree that extreme climate risks are neglected, and integrated catastrophic climate assessments are needed.

We also agree that the Intergovernmental Panel on Climate Change (IPCC) is not a perfect venue for assessing catastrophic climate risks. It is slow and its consensus decision-making tends to lead to lowest-common-denominator outcomes (3) and poses a challenge for extreme risk analysis. Yet, it offers legitimacy, credibility, and experience that few other bodies can match.

Every potential option has strengths and weaknesses. Craig and Ruhl’s proposal for “an independent, science-based governmental entity.… similar to the U.S. Geological Survey” would face key shortcomings in needing to establish a new organization and its international legitimacy would be questioned if it is tied to one government (or a few). In any case, such approaches are not mutually exclusive. We welcome efforts to establish processes for assessing extreme climate risks alongside an IPCC special report on catastrophic climate change.

We concur with Ruhl and Craig that risk assessment should include potential societal responses. Indeed, ‘vulnerability’ and ‘risk responses’ are two of the fundamental components of our third research strand on societal fragility. We do not underestimate the potential for social breakdown. Instead, we already provide an approach to soberly investigating it.

We disagree that anticipatory governance should involve nondemocratic development of policy responses, including publicly unpalatable and potentially draconian interventions such as forced relocation. Large-scale technocratic approaches have a track record of backfiring (4). Moreover, the need for open democratic policy responses has been a key point of agreement across most replies to Climate Endgame (5–7). Deliberative democracy is not just advantageous because it is open and inclusive but also because it appears to improve collective judgment (8, 9). There is little reason to believe that purely expert-driven processes would be more effective at addressing complex, controversial problems. Especially since these are issues of values, not solely technical challenges, and deliberative democracy already involves expert elicitation and input.

Anticipatory research should underpin anticipatory governance. This can help avoid the misuse of emergency powers which have underpinned a despotic drift toward more autocratic governance, such as the transition from the Roman Republic to Empire (10). Rather than relying on opaque, reactive, and far-reaching emergency powers, we should use crises as an opportunity to have more transparent, accountable, and democratic governance (11).

However, it is worth noting that this discussion of policy responses is related to, yet separate from, catastrophic climate risk assessments.

We fully endorse Ruhl and Craig’s sentiment that researching catastrophic climate change scenarios will need to be an interdisciplinary endeavor. Our research agenda covers areas that would require cooperation between archaeologists, anthropologists, epidemiologists, as well as Earth System and political scientists.

Understanding extreme climate risks is an interdisciplinary endeavor that needs to consider social breakdown. Yet, both the research and responses need to be legitimate and democratic.
